# Documentation of the partograph in assessing the progress of labour by health care providers in Malawi’s South-West zone

**DOI:** 10.1186/s12978-017-0401-7

**Published:** 2017-10-23

**Authors:** Chrispin Mandiwa, Collins Zamawe

**Affiliations:** 1 Ministry of Health, South-West Zone Health Support Office, P.O. Box 3, Blantyre, Malawi; 2 Institute for Global Health, University College London, London, UK; 3 Malawi Health Sector Program (DFID Project), Lilongwe, Malawi

**Keywords:** Partograph, Obstructed labour, Documentation, Malawi

## Abstract

**Background:**

There is some evidence that appropriate use of partograph in monitoring the progress of labour could decrease delivery related complications. The documentation of parameters of partographs is however, poorly understood. The aim of the present study was to determine the extent to which health care workers are making use of the partograph in monitoring the progress of labour through checking the documentation of the parameters of the partographs.

**Methods:**

A hospital-based descriptive study involving retrospective review of partographs for births that occurred in 2016 was conducted in Malawi’s South**-**West zone. A total of 1070 partographs that were used to monitor labour in two public hospitals were reviewed to determine the documentation of the parameters of partographs and descriptive statistics were computed using statistical package for the social science software version 22.0.

**Results:**

Of the total 1070 partographs reviewed, 58.6% (*n* = 627) of the partographs had no recording of maternal blood pressure and 65.3% (*n* = 699) of the partographs had no temperature documentation. Moulding was not recorded in 25.4% (*n* = 272) of the partographs, foetal heart rate was not recorded in 14.9% (*n* = 159) of the partographs and descent of the foetal head was not recorded in 12.0% (*n* = 128) of the partographs.

**Conclusion:**

There is poor documentation of vital parameters of the partographs. This suggests insufficient monitoring of the progress of labour, which may lead to adverse pregnancy outcomes. To improve the accurate documentation of parameters of the partograph, there is a need to understand the problem and provide tailor-made solutions to address them and ultimately improve pregnancy outcomes. In the meantime, in-service refresher courses on partograph use to health care workers need to be conducted regularly. Supportive supervision to obstetric care providers and regular partograph audit could also improve documentation.

## Plain English summary

There is some evidence that appropriate use of partograph (or labour chart) in monitoring the progress of labour could decrease delivery related complications. The documentation of parameters of partographs is however, poorly understood. Therefore, we conducted this study to determine the extent to which health care providers are making use of the partograph in monitoring the progress of labour through checking the documentation of the parameters of the partographs in Malawi’s South-West Zone. We observed that in most of the partographs, the parameters necessary for monitoring the progress of labour were not documented as required. This suggests poor monitoring of the progress of labour, which may lead to adverse pregnancy outcomes. Future studies should examine the reasons for poor documentation of parameters of the partograph to come up with evidence-based recommendations.

## Background

Although the maternal mortality ratio (MMR) has dropped by approximately 45% in the last two decades, around 300,000 women continue to die each year globally due to avoidable pregnancy related complications [1]. Obstructed labour is a leading cause of maternal and neonatal mortality, especially in developing countries [2–4]. Globally, it is estimated that obstructed labour occurs in 5% of pregnancies and accounts for an estimated 8% of maternal deaths [5–7]. Obstructed labour may result in serious complications such as obstetric fistula, uterine rupture, puerperal sepsis and postpartum haemorrhage [8, 9]. A hospital-based study in Uganda reported that obstructed labour and its complications resulted in 26% of all maternal deaths and a maternal death audit conducted in Rwanda showed that 12.3% of maternal deaths were attributed to obstructed labour [6, 10]. A recent maternal death audit in Malawi has also uncovered that obstructed labour contributed to 28.5% of maternal mortality [11]. Having the knowledge and skills of using tools to recognize obstructed labour and intervene timely is a key part of averting maternal deaths.

The use of partograph (or labour chart) to monitor the progress of labour is one of the globally recognized tools for reducing maternal mortality [12]. The partograph was designed by Friedman in 1954 and further improved by Philpot and Castle who introduced the alert and actions lines to facilitate interventions during labour [13]. When used appropriately, a partograph can help health care providers identify obstructed labour and know when to take appropriate actions to avoid complications. A World Health Organization (WHO) study in South East Asia involving 35,484 women found that using a partograph contributed to reduced (a) prolonged labour from 6.4% to 3.4%, (b) need for augmentation of labour with oxytocin from 20.7% to 9.1%, (c) occurrence of caesarean sections from 9.9% to 8.3%, and (d) intrapartum stillbirths from 0.5% to 0.3%. Based on these findings, in 1994, the WHO declared universal use of the partograph in all settings in monitoring labour to help identify abnormal progress and provide timely intervention when required [14]. Since then, a partograph has been one of the core labour management tools for prevention of maternal mortality and morbidity.

In Malawi, the Ministry of Health adopted this tool for labour management in 1970s [15]. Nevertheless, despite the composite partograph being in use for over 45 years, there are continuing maternal deaths and injuries resulting from obstructed labour in the country. A recent Demographic Health Survey (DHS) estimates maternal mortality ratio to be 439 per 100,000 live births and the country is one of those classified by WHO to have made no progress towards reducing maternal mortality between 1990 and 2015 [16, 17]. This raises the question about the utility of partographs and to date little is known about how the health care providers in Malawi document and make use of the partographs. Elsewhere, studies have identified a high proportion of incomplete partographs, which may limit the impact of the tool [18–20]. The aim of this study was to determine the extent to which health care providers are making use of the partograph in monitoring the progress of labour through checking the documentation of the parameters of the partograph. We hoped to identify the extent to which partographs are used to make clinical decisions.

## Methods

### Study setting

For administrative reasons, the Ministry of Health in Malawi divides the country into five zones. These are South-West, South-East, Central-West, Central-East and Northern-Zone. This study was conducted in South-West zone, which is the largest zone and comprises seven districts namely Blantyre, Chikwawa, Chiradzulu, Mwanza, Neno, Nsanje and Thyolo. The study was specifically undertaken at the two district hospitals in Thyolo and Chiradzulu, which were selected from a pool of all district hospitals in the zone using a simple random sampling method.

### Study design

This was a hospital-based descriptive study involving retrospective review of partographs for births that occurred in 2016. We examined the documentation of the parameters of partographs that were used to monitor the progress of labour at two selected district hospitals. We reviewed the documentation of parameters of the partographs, such as the foetal heart rate, moulding, descent of the foetal head and cervical dilatation that are crucial for making timely clinical decisions regarding childbirth care [21].

### Sampling procedure

At each hospital, we sampled partographs for 2 months from a list of 12 months for the year 2016. The months selected for Thyolo were April and July while for Chiradzulu it was February and August.

In total, 1386 partographs (714 from Chiradzulu hospital and 672 from Thyolo hospital) were retrieved from the medical records for the stated periods. We excluded partographs for pregnant women who had files showing conditions like intrauterine foetal death (IUFD), two previous caesarean sections, breech presentation and those who were planned for elective caesarean section. Since the vaginal examination is contraindicated in conditions like cord prolapse and antepartum haemorrhage (APH), we also excluded all partographs recorded cord prolapse and APH. Using these criteria, a total of 316 partographs were excluded and the final sample size was 1070.

### Data collection tool and procedure

A checklist tool was developed and used to extract data from the partographs. The tool assessed the components of the partograph to determine whether they had been monitored and documented per the national standard protocol developed by the reproductive health unit under the Ministry of Health in collaboration with ‘Save the Children International’. This national standard protocol states that descent of the foetal head, uterine contractions, maternal blood pressure, respirations and pulse rate should be monitored every hour, moulding and cervical dilatation every 4 h, temperature every 2 h and foetal heart rate every 30 min. The documentation status of the parameters was defined based on the time interval of documentation. Each parameter recorded on partograph not meeting any of the accepted time interval or with parts misplaced/missing or inadequate was judged as partially done, if no information was documented on the parameters of the partograph it was regarded as not done, and if all the criteria were satisfied for each parameter on the partograph, the documentation was considered as fully done.

All the partographs were independently scrutinized by two qualified clinicians using the checklist to determine the documentation of foetal heart rate, moulding, descent of the foetal head, cervical dilatation, uterine contractions, state of membranes, blood pressure, temperature and action line crossed/not crossed. The checklist also assessed the cadre of the health care worker charting the partograph (clinician, qualified midwife/nurse, students) as well as the gravidity of the woman (primgravida, multigravida). Furthermore, the checklist also assessed the recordings of the parameters of the newborn baby which included time of delivery, mode of delivery, foetal outcome, the sex of the baby, weight of the newborn and APGAR score of the baby.

### Data management and analysis

Partographs for the selected months were retrieved by hospital data clerks and this was done twice by different people to ensure that all available files were included. Each partograph was separately reviewed by two clinicians and the completeness of the parameters was recorded on a standard checklist form that was specifically developed for this study. Disagreements were resolved through discussion. The checklist forms were checked for completeness and consistence and then cleaned, coded and entered into Microsoft Excel spreadsheet. Once the entry was completed, the data was exported to SPSS statistical software (version 22.0, IBM, Inc.) for analysis. Descriptive statistics were computed to assess the documentation status of partograph forms. Frequency distributions and a graph were used to describe the variables of the study.

### Ethical approval

Permission to conduct this study was obtained from the hospital management of the two health facilities and matrons of each selected hospital were contacted before the commencement of the study. The names of pregnant women and health care providers on the partographs were not extracted and as such the data remained anonymous.

## Results

Of the 1070 partographs reviewed, 56.1%, 25.0% and 3.5% were documented by qualified nurses/midwives, students and clinicians, respectively. Most of the partographs (53.5%) were for multigravidas.

Table 1 presents the recording of parameters of maternal and foetal condition on the partograph. Foetal heart rate was not recorded in 14.0% of the partographs while 54.7% of the partographs were documented partially on foetal heart rate. Similarly, moulding and descent of the foetal head were not recorded in 25.4% and 12.0% of the partographs, respectively. A considerable proportion (75.5%) of the partographs was fully documented on the dilatation of the cervix and about half of the partographs partially documented uterine contraction. On maternal vital signs, 58.6% of the partographs had no recordings of maternal blood pressure and temperature was not documented in 65.3% of the partographs. The status of membranes was recorded in 77.1% of the partographs and an action line was crossed in very few partographs (8.1%).

**Table 1:** Recording of parameters of maternal and foetal condition on the partograph in South-West zone Malawi

Parameter	Frequency(*n* = 1070)	Percentage
Foetal heart rate		
Not recorded	159	14.9
Partially recorded	389	54.7
Fully recorded	326	30.5
Moulding		
Not recorded	272	25.4
Partially recorded	328	30.7
Fully recorded	470	43.9
Descent of foetal head		
Not recorded	128	12.0
Partially recorded	450	42.0
Fully recorded	492	46.0
Cervical dilatation		
Not recorded	86	8.0
Partially recorded	176	16.4
Fully recorded	808	75.5
Uterine contraction		
Not recorded	125	11.7
Partially recorded	529	49.4
Fully recorded	416	38.9
Action line crossed		
Yes	87	8.1
No	983	91.9
Blood pressure		
Not recorded	627	58.6
Partially recorded	310	29.0
Fully recorded	133	12.4
Temperature		
Not recorded	699	65.3
Partially recorded	240	22.4
Fully recorded	131	12.2
Status of membranes		
Recorded	825	77.1
Not recorded	245	22.9

We also examined the recording of baby information soon after birth (Table 2). From the reviewed partographs, 97.4% recorded the delivery time of the baby, 95.6% recorded the mode of delivery, 96.3% recorded foetal outcome, 97.4% recorded sex of the newborn, 97.0% recorded weight of the newborn and 98.2% recorded the APGAR score of the newborn.

**Table 2:** Recording of baby information after birth

Variable	Frequency	%
Delivery time		
Recorded	1042	97.4
Not recorded	28	2.6
Mode of delivery		
Recorded	1023	95.6.
Not recorded	47	4.4
Foetal outcome		
Recorded	1030	96.3
Not recorded	40	3.7
Sex of the newborn		
Recorded	1042	97.4
Not recorded	28	2.6
Weight		
Recorded	1038	97.0
Not recorded	32	3.0
Apgar score		
Recorded	1051	98.2.
Not recorded	19	1.8

**Fig. 1:**
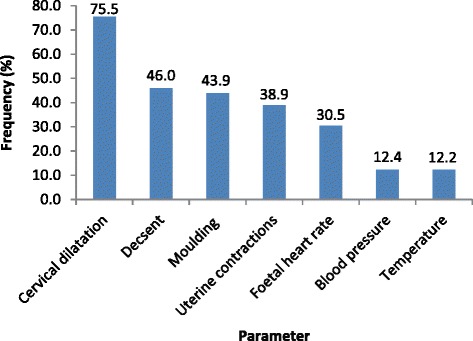
Proportions of partographs on which parameters were fully recorded

The proportion of partographs on which parameters were fully recorded to standard was assessed graphically. Cervical dilatation was the parameter that was recorded fully in most of the partographs while temperature and blood pressure were recorded fully in few partographs as shown in Fig. 1.

## Discussion

The findings show that health care providers did not accurately record all the parameters on the partograph to monitor the progress of labour as per the national guidelines in Malawi. We have observed that vital signs, such as temperature and blood pressure were not monitored at all in most of the partographs. Monitoring of maternal vital signs helps in assessing the general condition and detects any problems with the mother. For instance, checking of blood pressure may help to detect pre-eclampsia and eclampsia while temperature checking helps to identify fever, which indicates sepsis. Therefore, the importance of monitoring maternal vital signs in averting pregnancy related complications cannot be overemphasized.

Our observation that vital signs were not recorded in most partographs is similar to those reported by a study in Dar es Salaam hospitals, which found that blood pressure, temperature and pulse were not recorded in 47%–76% of the partographs [22]. Unavailability of equipment and lack of knowledge on the importance of the partograph by obstetric care providers could be part of the reasons for low documentation of the partographs, however, these need to be ascertained. For the time being, it could help if hospital managers make sure that all the required equipment for monitoring of maternal vital signs are available in all health facilities as well as regular supportive supervision for obstetric providers need to be conducted to ensure safety of the parturient women.

We noted that cervical dilatation was fully recorded in 75.5% of the partographs and this agrees with finding reported in Tanzania and Uganda where cervical dilation was recorded in 97.0% and 75.5% of the partographs, respectively [22, 23]. On the other hand, our finding is in contrast to a study conducted in Ethiopia where cervical dilatation was monitored to standard in only 32.9% of the partographs [19]. The discrepancies in the recording of cervical dilatation could be due to differences in sample size of the partographs reviewed and knowledge gap among obstetric care providers. This highlights the need for further studies to understand the reasons behind non-compliance.

The results indicate that foetal heart rate, descent of the foetal head and moulding were not adequately documented on the partograph and this suggests insufficient monitoring of the foetus. To improve foetal outcome, it is extremely important to monitor these parameters as it can help health care workers identify obstructed labour and intervene in a timely manner. Partial recording of these parameters on the partographs against the accepted standards was also observed in studies done in Tanzania and Kenya [22, 24]. This suggests that health care providers give less attention to these parameters, which might result into missed diagnosis of obstructed labour.

The documentation of baby information after birth was satisfactory as majority of the partographs recorded delivery time, mode of delivery, foetal outcome, weight of the baby and APGAR score of the newborn. This finding is consistent with the finding of a study conducted in Ethiopia in which all these parameters were also recorded appropriately [18]. This may suggest that health care providers find it easy to record these parameters since it is done once and after delivery as compared to the other parameters that need to be recorded at certain intervals.

We observed that over half of the sampled partographs were documented by qualified midwives while clinicians only documented a few. This finding is not necessarily surprising, as midwives constitute a bulk of skilled obstetric care providers in Malawi and are the ones who attend to pregnant women and clinicians are only involved when there are complications or when there is abnormal progress of labour. These findings fall in line with a recent study conducted in Cameroon which also reported that midwives constituted a higher proportional of health care providers using the partographs in monitoring labour [25].

This study had some limitations that must be considered in the interpretation and application of the results. First, the evaluation of partographs was retrospective, and some of important data were not available. Therefore, the retrospective study design prevented us from evaluating possible association between completion of partograph and obstetric outcomes as well as performing in depth descriptive analysis (e.g. partograph recording practices disaggregated with age or years of work experience). Second, the present study also used different and unequal time bands (months) for evaluating health care provider practices in the two hospitals which might not give a true reflection of health workers practice as one hospital might have a high staff attrition than the other. Partographs reviewed were collected from one zone and the findings may not be representative of the whole Malawi and thus, generalization of the findings should be made with caution. Finally, our study assessed only the documentation of the partographs to monitor the progress of labour and not whether partograph documentation was translated into labour management because filling the partograph does not necessarily mean actually using it to monitor the progress of labour. Notwithstanding, the findings provide a glimpse into current use of the partograph and lays the foundation of a larger study in the future.

## Conclusion

There is poor documentation of vital parameters of the partographs, which may be an indication for poor monitoring of labour by health care providers. Possible reasons for poor documentation of the partograph could be shortage of healthcare workers, complexity of the chart, knowledge and skill gap among health care workers on how to use the partograph and limited knowledge on the importance of the tool. As the focus of this study was on completeness of the partographs, future studies should ascertain reasons for non-compliance and provide evidence based recommendations. In the meantime, however, in-service refresher courses on partograph use to obstetric care providers through the continuing professional development (CPD) sessions and monitoring or audit of the partograph use, including recording and decision making could improve the documentation of parameters of the partograph. There is also a need to strengthen the ongoing supportive supervision to obstetric care providers so that all labours are monitored with the partograph.

## Abbreviations

APH: Antepartum haemorrhage
HMIS: Health management information system
IUFD: Intrauterine foetal death
MMR: Maternal mortality ratio
WHO: World health organisation
